# Persistent Hepatitis B Viraemia with Polymerase Mutations among HIV/HBV Co-Infected Patients on HBV-Active ART in KwaZulu-Natal, South Africa

**DOI:** 10.3390/v14040788

**Published:** 2022-04-10

**Authors:** Nokukhanya Msomi, Raveen Parboosing, Eduan Wilkinson, Jennifer Giandhari, Kerusha Govender, Benjamin Chimukangara, Koleka P. Mlisana

**Affiliations:** 1 Discipline of Virology, School of Laboratory Medicine and Medical Sciences, University of KwaZulu-Natal, Durban 4000, South Africa; parboosingr@ukzn.ac.za (R.P.); govenderk7@ukzn.ac.za (K.G.); chimukangarab@ukzn.ac.za (B.C.); 2 National Health Laboratory Service, Inkosi Albert Luthuli Central Hospital, Durban 4091, South Africa; 3 KwaZulu-Natal Research Innovation and Sequencing Platform (KRISP), School of Laboratory Medicine and Medical Sciences, College of Health Sciences, University of KwaZulu-Natal, 719 Umbilo Road, Durban 4001, South Africa; ewilkinson83@gmail.com (E.W.); jennifer.giandhari@gmail.com (J.G.); 4 National Health Laboratory Service (Academic Affairs, Research and Quality Assurance), Johannesburg 2131, South Africa; koleka.mlisana@nhls.ac.za

**Keywords:** HBV virological failure, HBV-persistent viraemia, HBV drug resistance, HIV/HBV coinfection

## Abstract

To understand the problem of persistent Hepatitis B virus (HBV) viraemia in HIV/HBV co-infected patients on HBV-active antiretroviral therapy (ART), we assessed the rate of HBV virological response in patients on HBV-active ART in KwaZulu-Natal, South Africa, and analysed factors associated with persistent HBV viraemia. One hundred and fifty eligible participants with a chronic HBV diagnosis, with or without HIV coinfection, were enrolled and followed up after 6 months. The HBV pol gene was sequenced by next-generation sequencing and mutations were determined using the Stanford HBVseq database. Logistic regression analysis was used to assess factors associated with HBV viraemia at 6-month follow-up. The mean duration of HBV-active ART was 24 months. Thirty-seven of one hundred and six (35%) participants receiving HBV-active ART for longer than 6 months had virological failure. Advanced immunosuppression with CD4+ cell counts <200 cells/μL was independently associated with persistent HBV viraemia, aOR 5.276 (95% CI 1.575–17.670) *p* = 0.007. A high proportion of patients on HBV-active ART are unsuppressed, which will ultimately have an impact on global elimination goals. Better monitoring should be implemented, especially in HIV-coinfected patients with low CD4+ cell counts and followed by early HBV drug-resistance testing.

## 1. Introduction

Chronic Hepatitis B Virus (CHBV) infection is a global health problem, and the World Health Organization (WHO) estimates that 257 million people are infected with HBV worldwide [1]. Human immunodeficiency virus (HIV) is another prevailing global health problem with ~37.7 million people living with HIV in 2020 [2]. Co-infection with both HIV and HBV is particularly prevalent in sub-Saharan Africa and is reported in up to 36% of HIV-infected individuals in the western and southern parts of the continent [3]. HIV/HBV co-infection is associated with poorer clinical outcomes, with reports of higher levels of HBV viraemia, frequent episodes of reactivation and more rapid progression of liver fibrosis and HBV-related hepatocellular carcinoma [4,5,6]. Liver disease-related mortality is also a leading cause of death in people living with HIV (PLWH) [7].

The availability of nucleos(t)ide analogues (NAs) that are active in the treatment of both infections has been important in managing co-infected patients, but continued lifelong treatment with NAs has its own challenges [8]. In patients with CHBV infection, mutant viruses may emerge as a result of selection pressure exerted by NAs and/or through immune evasion. These mutants form part of the quasispecies and may limit future therapeutic options due to cross-resistance, or may produce HBV vaccine escape mutants [9].

Although HBV is a DNA virus, it replicates via an RNA intermediate using a reverse transcriptase (RT) polymerase (pol) enzyme that has poor proofreading ability [10]. Under selective drug pressure, HBV polymerase develops spontaneous mutations that can become dominant and lead to treatment failure. Eight codons in the RT domains of Pol have been associated with primary resistance to NAs, namely, 169, 180, 181, 184, 202, 204, 236 and 250 [11]. Mutations at these codon positions include the catalytic region of the RT-polymerase enzyme with the tyrosine–methionine–aspartic acid–aspartic acid (YMDD) motif. Naturally occurring YMDD mutations in the polymerase catalytic region have been shown to exist in large proportions of patients with CHBV who are treatment-naïve [12]. This poses a potential risk of breakthrough viraemia once patients are started on treatment, with selection and archiving of multi-drug-resistant mutants [13].

The approved NAs for the treatment of CHBV include lamivudine (LAM), tenofovir disoproxil fumarate (TDF), tenofovir alafenamide fumarate (TAF), adefovir dipivoxil (ADV), entecavir (ETV) and telbivudine (LdT). Of these NAs, TDF and LAM are currently available for the treatment of HIV/HBV co-infected individuals in the South African public health sector. TDF/LAM-containing first-line ART is preferred for HIV/HBV co-infected individuals because of its superiority to LAM monotherapy [14]. However, HBV drug resistance (HBVDR) with virological failure remains a significant clinical problem due to the limited availability of treatment options. The goal of HBV treatment is to maintain virological suppression and delay progression of liver disease. Prospective studies from Europe have reported virological suppression with TDF-containing regimens at above 90% for over 4 years [15,16], with a multi-centre prospective cohort study conducted in Australia, the United States of America, and Thailand reporting suboptimal and persistent HBV viraemia in 44% of patients on TDF-containing ART [17]. HBV resistance to TDF is still not well-described [18], and clinical determinants and factors associated with persistent HBV viraemia in this scenario remain ill-defined.

Southern Africa is considered an HBV-endemic region, and infection with HBV is generally acquired in childhood before five years of age, whereas HIV infection occurs later in life, predominantly via heterosexual intercourse [19]. At the time of acquiring HIV and commencing dual active treatment, some patients would have been chronically infected with HBV since childhood. The accumulation of naturally occurring HBVDR-associated mutations in treatment-naïve patients is probable. The limited treatment options in patients who fail to achieve viral suppression with TDF/LAM-containing first-line treatment means that these patients are generally kept on sub-optimal therapy with further accumulation of HBVDR mutations and ongoing viral replication. Ongoing replication from the covalently closed circular DNA and persistent viraemia during therapy leads to the infection of new cells and progression of the disease [20].

The European Association for the Study of the Liver (EASL) defines virological response during NA therapy as undetectable HBV DNA by a sensitive polymerase chain reaction (PCR) assay that has a lower limit of detection of 10 IU/mL Primary non-response is defined as a less than one log_10_ decrease in serum HBV DNA after 3 months of therapy. Partial virological response is defined as a decrease in HBV DNA of more than 1 log_10_IU/mL but detectable HBV DNA after at least 12 months of therapy in compliant patients. HBV resistance to NAs is evidenced by the selection of HBV variants with amino acid substitutions that confer reduced susceptibility to the administered NA(s) [21]. The same definitions of virological failure are also affirmed by the WHO guidelines for managing persons with CHBV [22].

We sought to describe the rate of HBV viral suppression in a cohort of patients receiving HBV-active ART, characterize patterns of pre-existing HBV mutations and those mutations that emerge and evolve in HIV/HBV co-infected patients on HBV-active ART, and describe the clinical factors associated with HBV genomic heterogeneity in these patients.

## 2. Materials and Methods

### 2.1. Study Participants and Data Collection

This was a prospective cohort study where a total of 150 adult study participants were recruited from a communicable disease clinic at Edendale Hospital in Pietermaritzburg, KwaZulu-Natal, South Africa, between June 2017 and January 2019. Enrolment criteria was confirmed CHBV diagnosis based on HBsAg-positive result persisting beyond 6 months, with or without HIV coinfection. Once enrolled, the participants were seen at 2 time points at least 6 months apart. At the time of enrolment, a proportion of the patients were treatment-naïve for both HIV and HBV, and others were receiving HBV-active ART.

At study enrolment, clinical data collected from medical charts included demographics, prior and current anti-HIV and HBV-active ART, previous/present tuberculosis infection, and any significant HIV and liver-related illnesses. Laboratory data collected included liver function markers: alanine aminotransferase and aspartate aminotransferase; HBV serological markers: hepatitis B surface Ag (HBsAg), hepatitis B e antigen (HBeAg), hepatitis B surface antibody (anti-HBs); and hepatitis B core antibodies. Hepatitis A IgM, HCV antibody, HIV viral load (HIVVL), CD4 cell count, and previous HBV viral load (HBVVL) results were also collected. Blood samples were collected for HBV serology, HBVVL, and HIVVL at enrolment and for HBVVL only at the 6-months follow-up visit. Sequencing was performed on plasma samples with detectable HBVVL at enrolment and in those patients remaining viraemic at the 6-months follow-up visit.

### 2.2. HBV and HIV Quantification

HBV DNA quantification was performed using the COBAS AmpliPrep/COBAS TaqMan HBV V2 test on the automated COBAS AmpliPrep and COBAS TaqMan Analyzer (Roche Molecular Systems, Inc., Branchburg, NJ, USA), according to manufacturer’s instructions. The analytical measurement range of the assay is 20 to 1.7 × 10^8^ IU/mL, and the conversion factor between HBV copies/mL and HBV IU/mL is 5.82 copies/IU, using the WHO International Standard for Hepatitis B Virus DNA for Nucleic Acid Technology (NAT) Assays Testing (NIBSC 97/746). The assay’s lower limit of detection is 9IU/mL (95% confidence range 6.8–12 IU/mL). HIV was quantified on the COBAS AmpliPrep and COBAS TaqMan 6/8800 analyzer as well as on the Abbott m-Alinity analyzer (Abbott Laboratories, Abbott Park, IL, USA).

### 2.3. HBV Nested Polymerase Chain Reaction

All plasma samples with detectable HBV DNA by real-time quantitative PCR were further tested with a nested PCR targeting the surface/polymerase regions of HBV genome. DNA was extracted using the easyMAG automated extraction system (bioMérieux Marcy L’Étoile, France), and the surface/polymerase regions of HBV were amplified by nested PCR on a Veriti thermal cycler (Applied Biosystems, Life Technologies—Carlsbad, CA, USA). Details of primer sequences and cycling conditions are shown in Supplementary Table S1. Second-round PCR products were run on a 1% agarose gel and visualized on a BioRad UV transilluminator (BioRad Laboratories Inc. Hercules, CA, USA) to visualize an 800 bp product. The amplification of samples with lower viral loads (<500 IU/mL) was variable, but sequencing was attempted in all samples with a quantifiable HBVVL of >20 IU/mL.

### 2.4. PCR Purification and Sequencing

PCR amplicons were purified using the QiaQuick PCR Purification kit (QIAGEN, Hilden, Germany) and eluted in a final volume of 60 uL. Input DNA was quantified using Qubit dsDNA HS Assay system (ThermoFisher Scientific Inc, Waltham, MA, USA), diluted to 0.2 ng/uL, and library preparation was performed using the Nextera XT DNA Library Preparation Kit (Illumina, San Diego, CA, USA) according to the manufacturer’s instructions. In summary, library preparation involved kit-based enzymatic fragmentation of DNA, dual indexing of fragmented DNA using the Nextera XT Index kit (Illumina, San Diego, CA, USA), and purification of barcoded amplicons using AMPure XP beads (Beckman Coulter, Atlanta, GA, USA). Fragment quality was assessed using a LabChip GX Touch (Perkin Elmer, Waltham, MA, USA), with a final average fragment length of 350 bp obtained. Libraries were normalized to 4 nM and pooled at a final concentration of 10 pM. Libraries were spiked with 5% PhiX and sequenced on an Illumina MiSeq instrument using a MiSeq Reagent Nano kit v2 500 cycles (Illumina, San Diego, CA, USA).

### 2.5. Sequence Analysis

Paired-end raw sequence reads were assembled using GenomeDetective, a web-based viral assembly tool (https://www.genomedetective.com accessed 1 July 2020) [23]. Binary alignment files (BAMs) and consensus contigs were obtained for each sample and were further analyzed in Geneious Prime (https://www.geneious.com accessed 1 July 2020). Highly variable regions on the terminal ends of the consensus sequences were verified by querying BAM files.

Good quality consensus sequences were analyzed using the jumping profile Hidden Markov Model HBV genotyping tool (http://jphmm.gobics.de accessed 1 July 2020) [24] and by phylogenetic inference. Five hundred and thirty-three HBV reference whole genomes covering all major genotypes (A–J) and subgenotypes (e.g., A1–A7) were downloaded from GenBank and aligned against individual polymerase (pol) sequences using MAFFT [25] in Geneious Prime (Biomatters Limited, Auckland New Zealand). The best nucleotide substitution model for each alignment was identified through the ModelFinder package [26] embedded in IQTree [27]. Maximum likelihood (ML) phylogenies were inferred in IQTree v 2.0.6 with 1000 bootstrap replicates [28]. The consensus ML trees and bootstrap trees were then used to infer for splits in the phylogenies using BOOSTER and the transfer bootstrap equilibrium (TBE) method [29]. Phylogenies were annotated for illustration using ggtree package in R [30].

For patients remaining viraemic at six-month follow-up, a second sequence for the pol gene was performed. Pairwise comparison between baseline and follow-up sequences were performed in Geneious Prime to identify all synonymous and non-synonymous (ds and dn) mutations. Finally, all RT sequences in FASTA format were run on the Stanford HBVseq database for detection of known HBV nucleos(t)ide analogue associated mutations and resistance (https://hivdb.stanford.edu/HBV/HBVseq/development/hbvseq.pl?action = showSequenceForm accessed 12 December 2021).

All sequences that were analyzed in this study have been uploaded on GenBank, and accession numbers are listed in Supplementary Materials Table S3. https://www.ncbi.nlm.nih.gov.

### 2.6. Statistical Analysis

SPSS Statistics software (Version 26; IBM-New York, NY, USA) was used to calculate unadjusted and adjusted odds ratios, based on a logistic regression model of factors associated with HBV viraemia at 6-month follow-up. The following baseline variables were tested: sex, age, TB history, the level of immunosuppression as determined by CD4+ count, ALT, HIVVL at first visit, and the duration of HBV-active ART at time of enrolment.

## 3. Results

Of the 150 participants enrolled in the study, 64% were males, and the mean age was 38 years (SD: 9.2). All participants were of African ethnicity. All except one participant (99%) had HIV/HBV coinfection. At the time of enrolment, 143 (95%) were already on an HBV-active ART regimen containing TDF + LAM, and the median duration on TDF-containing ART was 2 years (range 0–11). Of the 143 participants, 23 (16%) were on TDF + LAM for ≤6 months, while 106 (74%) were on TDF + LAM for longer than 6 months at enrolment. Of these, 106 participants were on treatment for longer than 6 months and 69 (65%) had achieved HBV virological suppression. Six participants were on 3TC monotherapy for HBV due to renal impairment precluding the use of TDF. Participants’ demographic and clinical characteristics at baseline are summarized in Table 1.

**Figure 1:**
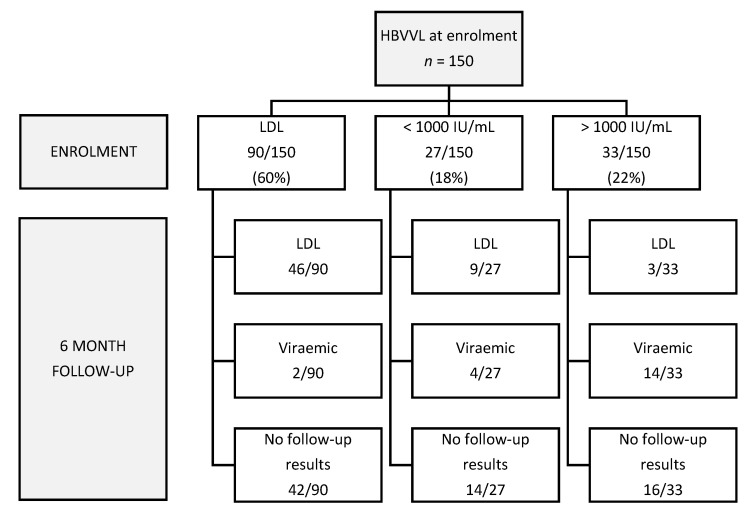
Flow chart: HBVVL at baseline and follow-up.

Of the 150 participants, 56 (37.3%) had complete HIV viral suppression at baseline with HIVVL below the level of detection. HIV virological failure (defined as HIVVL ≥ 1000 copies/mL) was diagnosed in 51 (34%) participants, and partial HIV suppression was found in 43 (28.7%) participants as outlined in Table 1. Seventy-three participants (49%) were followed up 6 months later, and 54/73 (74%) were virologically suppressed for HBV at 6 months, while 19/73 (26%) had persistent HBV viraemia (defined as a detectable viraemia). Figure 1 shows the pattern and distribution of HBVVL at enrolment and at the 6-month follow-up visit.

Younger participants (i.e., aged 26–35 years) with CD4+ counts < 200 cells/mm^3^, participants with baseline HIVVLs >1000 copies/mL, and those on HBV-active ART for >6 months had higher odds of persistent HBV viraemia. However, only advanced immunosuppression (CD4+ cell count < 200 cells/mL) remained statistically significant in the adjusted model (Table 2).

### Next Generation Sequencing and Phylogenetics

Of the 37 samples from participants that had detectable viraemia at enrolment and at 6-month follow-up, 33 were successfully sequenced. Of the sequenced samples, 88% had ≥Q30 quality threshold and were included in final analysis. These had 35,000 average number of reads per sample.

In total, 15/33 (45.5%) sequences were from patients who have received treatment for 6 months or less, and 18/33 (54.5%) sequences were from patients who were relatively treatment-experienced at the time of enrolment.

**Figure 2:**
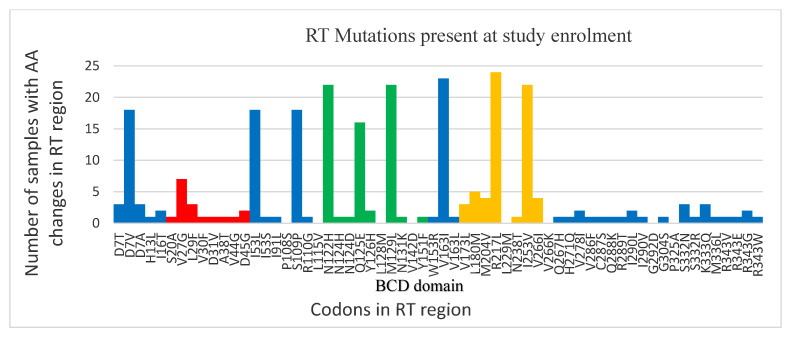

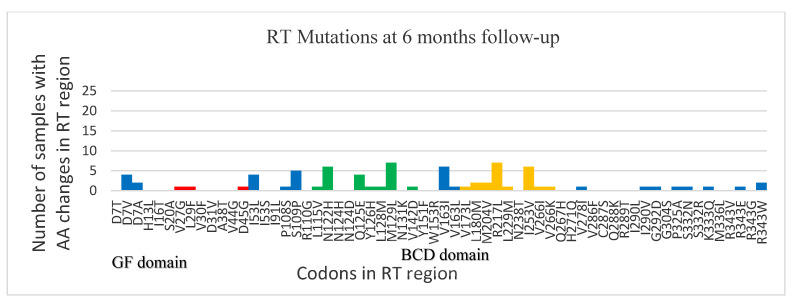
Patterns of HBV RT mutations observed at first and second visit.

Of the 33 sequences, 26 (79%) sequenced had at least one HBV drug resistance mutation detected, of which 5 (19%) were primary and secondary mutations in the B and C catalytic domains of the RT region of pol. The most common mutations included a combination of V173L, L180M, M204V, which are associated with ETV, 3TC and LdT resistance. Figure 2 and Supplementary Materials Table S2 summarize patterns and distribution of mutations detected in the sequenced samples at baseline and at 6-month follow-up.

There was a similar pattern of polymorphic mutations in most sequences. When describing the kinetics of HBV drug resistance emergence, Zoulim et al. show that, as the viral quasispecies evolve, polymorphic mutations herald primary and secondary resistance mutations, which initially lead to partial response and, subsequently, to overt resistance [11].

**Figure 3:**
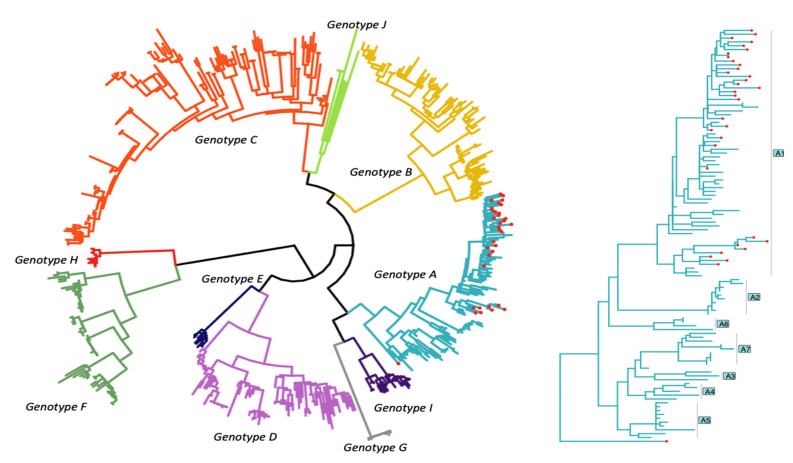
Maximum likelihood phylogenetic tree showing ten HBV genotype references and an expansion of genotype A with study sequences represented by red dots. Booster support values (>0.95) for major branches.

Notably, three participants had drug resistance mutations after receiving HBV-active ART for <6 months, suggesting pre-existing mutations. All sequences belonged to either the sub-genotype A1, except one sequence whose subgenotype could not be accurately classified, as shown in Figure 3.

Red-shaded bars show mutations in the G and F domains of the reverse transcriptase (RT) region, yellow bars show mutations in the B, C and D domain of RT, which contain catalytic sites, whilst the green bars show the area where changes in pol result in s-gene mutations in overlapping frame

## 4. Discussion

In this study, we found a large proportion (35%) of participants with persistent HBV viraemia on long-term TDF + LAM HBV-active ART. This is concerning, considering that this treatment has a relatively high genetic barrier compared to other treatment regimens used for managing CHBV. A multi-centre study conducted in Australia and Thailand also reported similarly high rates of persistent viraemia in HIV/HBV co-infected patients on TDF-containing ART [31]. In a similar setting of the southern African countries of Zambia and South Africa, HBV viral suppression was achieved in only 61.5% of LAM-treated and 71.4% of TDF-treated patients [14], suggesting that persistent viraemia drives quasispecies diversity with the selection and accumulation of drug resistance mutations in the presence of drug pressure [11]. This is a cause of concern because residual and persistent HBV viraemia have both been associated with hepatocellular carcinoma in chronic hepatitis B patients receiving antiviral therapy [32].

Reasons for the non-suppression of HBV viraemia in patients on HBV-active ART remain unclear. In this study, severe immunosuppression as indicated by a CD4+ count below 200 cells/mL was an independent factor associated with HBV viraemia. In other studies, which looked at similar patient profiles, low CD4+ counts as well as HBeAg positivity were associated with HBV viraemia upon treatment [17,33], a trend also observed in this study. This observation strengthens the rationale for the early treatment of HIV in HBV co-infected individuals, irrespective of immune-suppression level, to achieve better treatment outcomes. South Africa has a syndemic of HIV, HBV, and tuberculosis (TB). In our cohort, a significant proportion (27%) of participants had a history of TB as well. Although a history of TB was found to have 1.7-fold odds of association with persistent HBV viraemia at follow up, this was not statistically significant during multivariate analysis. In another South African large-cohort study, which studied factors associated with the incidence and persistence of HBV infection in patients receiving HIV care, a history of TB was found to be associated with a higher incidence of HBV infection [34].

Although mutations at codons rt194 and rt236, which have been associated with the alkyl phosphonate resistance pathway conferring resistance to ADV and TDF resistance, were not detected in this cohort, primary and secondary resistance mutations at codon rt173, rt180, and rt204 were detected in 19.2% of sequences at study enrolment. The rtV173L, rtL180M, and rtM204V mutations are not only associated with LAM, ETV, and LdT resistance, but have also been shown to enhance replication competence [35]. Another South African study reported a similar pattern of rtV173L + rtL180M + rtM204V and rtV214A in 10% of treatment-naïve HIV/HBV coinfected patients [36]. Sheldon et al. reported that the LAM-associated mutations rtL180M and rtM204V resulted in a 5.7-fold decrease in the HBV susceptibility to TDF, with phenotypic analyses showing that the rtA194T mutation, combined with rtL180M and rtM204V, had over a 10-fold increase in the IC50 for TDF compared with the wild type in HIV/HBV co-infected individuals [37]. This could explain the high rate of persistent viraemia observed in this cohort of HIV/HBV coinfected individuals on HBV-active ART. None of the sequences exhibited the quadruple CYEI mutation at positions rt106, rt126, rt134, and rt269, which have been recently shown to increase the amount of TDF required to inhibit HBV by 15.3-fold in IC50 and 26.3-fold in IC90, even though the majority of our patient cohort were on TDF for longer than 2 years. Notably, the CYEI mutation reportedly occurred in combination with rtV173L + rtL180M + rtM204V to cause TDF resistance [38].

The rtV173L + rtL180M + rtM204V triple mutant combination, which was observed in two participants at enrolment and persisted at follow up in one participant, has been shown to cause amino acid changes in the overlapping surface gene, resulting in reduced anti-HB binding [9,39]. This triple mutant has been reported with higher frequency in patients infected with HBV genotype A, and who are also HIV co-infected [40]. This was notably evident in this study, where all sequences clustered around genotype A1 and were mostly HIV co-infected. None of the participants with the triple mutant combination had evidence of surface antibodies on serological testing, suggesting possible immune escape. The bigger and most significant public health impact is not only the potential transmission of HBV drug-resistant mutants, but also vaccine-resistant variants [41]. However, our findings suggest that non-classical mutations may be associated with TDF failure in patients with HIV/HBV infection.

When describing the kinetics of drug resistance emergence, Zoulim and Locarnini describe that, prior to virological breakthrough and overt resistance, a significant proportion of genomes harbor polymorphisms which precede the emergence of dominant resistant variants [11]. This is also observed in the sequences of viraemic patients from our cohort showing high rates of polymorphisms in the A, B, C, D, F, and G domains of the RT region of pol. The exact role of these polymorphisms has not been fully explored in this study but could play a role in the observed persistent viraemia, resulting in reduced drug sensitivity and preceding the selection of known drug-resistance mutations. Evidence that polymorphisms may reduce susceptibility to TDF-based HBV treatment is emerging [42]. Other studies reporting a high frequency of complex mutational patterns in NA-experienced patients speculate on the potential role of these patterns in drug resistance [43] and disease progression [44], but further prospective studies are still needed to conclusively link clinical outcomes.

All sequences from this study cohort clustered around genotype A1 except for one that was not classified. This is similar to other South African reports showing that, in HBV/HIV co-infected patients, 97% of the HBV isolates clustered around the subgenotype A1 and 3% belonged to subgenotype D3 [36]. Genotype A1-infected individuals have been shown to have higher levels of liver damage and a higher risk of developing hepatocellular carcinoma [45].

A limitation to our study was the problem of loss to follow-up, as the recruitment site down-referred a lot of participants to local clinics for long-term care. This compromised participant follow-up.

In conclusion, high rates of persistent viraemia and poorer virological control of HBV, even on HBV-active ART, remains a challenge and creates a reservoir for the transmission of HBV variants. When HIV drives the viral replication of HBV in chronically infected individuals, the variants that arise do not only cause drug resistance, but may result in immune and vaccine escape, threatening the gains of the vaccination programme. Further research with phenotypic studies is needed to determine the impact of persistent viraemia in patients receiving high genetic-barrier treatment such as TDF, to inform better dosing strategies or regimen combinations for better treatment outcomes. Clarion calls have been made for the reprioritization of HIV/HBV management in South Africa [46] and sub-Saharan Africa [19] because of the unique challenges created by the syndemic of HIV and HBV in these parts of the world. Therefore, in response to these calls and the WHO strategy to eliminate viral hepatitis as a public health problem by 2030 [47], this study highlights the drivers of HBV infection in settings where HIV is a major confounder.

## Figures and Tables

**Table 1:** Demographic and clinical characteristics of participants at study enrolment.

Variables	Total n = 150
Gender, n (%)	
Male	96 (64.0)
Female	54 (36.0)
Age group (years), n (%)	
<18–25	14 (9.3)
26-36	50 (33.3)
36-45	56 (37.3)
46-61	29 (19.3)
History of TB status at enrolment, n (%)	
Yes	41 (27.3)
No	107 (71.3)
Unknown	2 (1.3)
CD4+ count at enrolment (cells/mm^3^), n (%)	
<200	64 (42.7)
201–500	54 (36)
>500	27 (18)
Unknown	5 (3.3)
HIVVL (copies/mL), n (%)	
LDL	56 (37.3)
<1000	43 (28.7)
>1000	51 (34)
HIVVL log_10_ median	3.27
HIVVL log_10_ SD	1.43
HIVVL log_10_ mean	3.37
HBV Viral load at enrolment (IU/mL), n (%)	
LDL	90 (60)
1–1000	27 (18)
1001–10,000	10 (6.7)
>10,000	23 (15.3)
HBVVL Log_10_ median	3.42
HBVVL Log_10_ SD	2.17
HBVVL Log _10_ mean	3.95
ALT, n (%)	
normal	87 (58)
2–5 × ULN	53 (35.3)
>5 × ULN	7 (4.7)
Unknown	3 (2.0)
HBV serological markers, proportion (%)	
HBsAg positive	135/145 (93)
HBeAg positive	71/143 (49.7)
Anti-HBc (total) positive	129/145 (89)
Anti-sAg titres	
>10 mIU/mL	8/145 (5.5)
<10 mIU/mL	137/145 (94.5)
HBV active ART, n (%)	
TDF + LAM	143 (95.3)
TDF only	1 (0.7)
LAM only	6 (4)
Duration of TDF + LAM at enrolment, n (%)	
≤6 months	37 (24.7)
>6 months	106 (70.6)
Unknown	7 (4.7)

LDL, lower than detection limit; ALT, Alanine aminotransferase; ULN, upper limit of normal; ART, antiretroviral treatment; HBVVL, HBV viral load; HIVVL, HIV viral load; TB, tuberculosis; TDF, tenofovir disoproxil fumarate; LAM, lamivudine.

**Table 2:** Factors associated with HBV viraemia at follow up.

Factor	Unadjusted OR (95% CI)	*p*-Value	Adjusted OR (95% CI)	*p*-Value
Gender (ref: female)				
Male	0.953 (0.483–1.880)	0.890	0.959 (0.429–2.142)	0.919
Age group (years) (ref: age ≥ 35)				
18–25	1.357 (0.439–4.192)	0.596	1.504 (0.371–6.102)	0.568
26–35	2.205 (1.078–4.512)	0.030	1.936 (0.849–4.413)	0.116
History of TB at enrolment (ref: No TB)				
Yes	1.390 (0.672–2.877)	0.374	1.747 (0.692–4.406)	0.237
CD4+ count (cells/mm3) (ref: ≥500)				
<200	5.213 (1.762–15.422)	0.003	5.276 (1.575–17.670)	0.007
201–500	2.497 (0.817–7.629)	0.108	2.756 (0.821–9.251)	0.101
HIVVL at baseline (copies/mL) (ref: <1000)				
>1000	2.250 (1.128–4.489)	0.021	2.014 (0.936–4.336)	0.073
ALT at baseline (ref: ALT ≤ 40IU/mL)				
ALT > 40 IU/mL	0.895 (0.463–1.730)	0.742	0.870 (0.411–1.842)	0.716
Duration of HBV-active ART (ref: <6 months)				
>6 months	0.437 (0.211–0.903)	0.025	0.465 (0.189–1.141)	0.094

ART, antiretroviral treatment; OR, odds ratio; TB, tuberculosis; HIVVL, HIV viral load; ALT, alanine transaminase.

## Data Availability

Not applicable.

## Supplementary Materials

The following supporting information can be downloaded at https://www.mdpi.com/article/10.3390/v14040788/s1: Table S1: PCR primers for HBV pol gene amplification and cycling conditions, Table S2: Patterns of mutations selected over time in patients on TDF + LAM, and Table S3: HBV Pol Sequences and accession numbers.
