# Supplementary material for: Persistent Hepatitis B Viraemia with Polymerase Mutations among HIV/HBV Co-Infected Patients on HBV-Active ART in KwaZulu-Natal, South Africa
Source: Viruses. 2022 Apr 10;14(4):788. doi: 10.3390/v14040788 (PMC9026734; doi:10.3390/v14040788)
Supplement: Supplementary file 1 [file viruses-14-00788-s001.zip › viruses-1583424-supplementary.pdf]

Supplementary Data

Supplementary Table S1 PCR primers for HBV *pol* gene amplification

PCR	Primer name	Primer sequence	Codon position	Amplicon size
First round	HBV 3	CGTTGCCKDGCAACSGGGTAAAGG	1140-1163	1000bp
	Z0404	AGCCCTCAGGCTCAGGGCATA	3085-3105	
Second round	POLF	TCATCCTCAGGCCATGCAG	3198-3217	800bp
	M5877	GACACACTTTCCAATCAATNGG	970-991	

PCR, polymerase chain reaction

Cycling conditions

First round PCR	Second round PCR
94°C 2 Minutes<br><br>94°C 30 Seconds }<br>55°C 30 Seconds } 35 Cycles<br>72°C 1 Minute }<br><br>72°C 2 Minutes<br>4°C HOLD	94°C 2 Minutes<br><br>94°C 30 Seconds }<br>50°C 30 Seconds } 30 Cycles<br>72°C 1 Minute }<br><br>72°C 6 Minutes<br>4°C HOLD

Supplementary Table S2 Patterns of mutations selected over time in patients on TDF + LAM

**Patient ID**	**HBVVL (IU/ml)**	**Duration on Rx (months)**	**Mutations in the RT region**	**Stanford HBVseq database resistance associated mutations**	**Resistance profile**
**RT mutation patterns at study enrolment**					
HBV10	967	8	D7T, N122H, V163I, **L180M,M204V**, R217L, N238T, I253V, V278I, V286F, C287S, Q288K, R289T,I290V	**L180M + M204V**	LAM: R<br>ADV: S<br>ETV: I<br>TDF: S
HBV18	2 451	42	D7V, V27G, I53L, S109P, N122H, M129L, V163I, R217L, I253V, V266K		
HBV23	6 772	7	D7V, 153L, S109P, N122H, Q125E, M129L, V163I, R217L, I253V		
HBV26		17	R217L, I253V,		
HBV33	27 016	12	D7A, V27G, L29F, N122H, M129L, V163I, R217L, I253V, V266I		
HBV34	9 918	2	D7A, I53S, I91L, N124H, Y126H, N131K,Y151F, W153R, V163I, V266I, Q267H,H271Q		
HBV47	>170000000	0	D7V, I53L, S109P, N122H, M129L,V163I, R217L, 1253V, V266I, S332N,K333Q		
HBV57	352 781	0	D7V, I53L, S109P, N122H, Q125E, M129L, V163I, R217L,		
HBV67	2 662	29	D7A, H13L, I16T, Y126H, M129L, V163I, L180M, M204V, R217L, I253V	**L180M + M204V**	LAM: R<br>ADV: S<br>ETV: I<br>TDF: S
HBV70	111	0	D7V, V30F, D31V, 153L, S109P, N122H, Q125E, M129L, V163I, V173L, L180M, R217L, I253V, G304S	**V173L + L180M**	LAM: R<br>ADV: S<br>ETV: R<br>TDF: S
HBV72	113 217 751	30	D7V, I53L, S109P, N122H, Q125E, M129L, V163I, R217L, I253V, S332N		
HBV85	2 597	3	D7T, N122H, M129L, V163I, R217L, I253V, V278I		
HBV87	3 525 565	0	D7T, V27G, L29F, I53L, S109P, R110G, N122H, Q125E, M129L, V163I, R217L, I253V		
HBV92	>170000000	0	D7V, I53L, S109P, N122H, Q125E, M129L, V163I, R217L, I253V, M336L		
HBV103	>170000000	2	D7V, I53L, S109P, N122H, Q125E, M129L, V163I, **V173L, L180M, M204V**, R217L, I253V, R343W	**V173L + L180M + M204V**	LAM: R<br>ADV: I<br>ETV: R<br>TDF: S
HBV110	118 093	1	D7V, I53L, S109P, N122H, Q125E, M129L, V163I, R217L, I253V		
HBV111	3 528	5	D7V, V27G, I53L, S109P, N122H, N124D, Q125E, M129L, V163I, R217L, I253V, I290L, R343G		
HBV112	7 556	1	D7V, I16T, S109P, N122H, Q125E, M129L, V163I, R217L, I253V		
HBV130	738 161	0	D7V, V27G, I53L, S109P, N122H, Q125E, M129L, V163I, R217L, I253V, I290L, R343E		
HBV134	136	24	D7V, V27G, A38T, D45G, I53L, S109P, N122H, Q125E, M129L, V163I, R217L, 1253V		

HBV136	>170000000	15	D7V, V27G, I53L, S109P, N122H, Q125E, M129L, V163L, **V173L, L180M, M204V**, R217L, I253V	**V173L + L180M + M204V**	LAM: R<br>ADV: I<br>ETV: R<br>TDF: S
HBV139	262	9	D7V, I53L, S109P, N122H, M129L, V163I, R217L, I253V, V266I, S332N, K333Q, R343G		
HBV144	110 074	1	D7V, I53L, S109P, N122H, Q125E, M129L, V163I, R217L, I253V, R343V		
HBV145	>170000000	30	D7V, 153L, S109P, N122H, M129L, V163I, R217L, I253V		
HBV146	349 663	0	D7V, V27G, L29F, S20A, V44G, D45G, I53L, N122H, Q125E, M129L, V163I, R217L, S332R, K333Q		
HBV150	19 199	92	D7V, I53L, S109P, N122H, Q125E, M129L, V163I, R217L, I253V		
**Follow up mutation patterns**					
HBV18_2	33 399	48	D7V, V27G, L29F, D45G, I53L, S109P, N122H, M129L, V163I, R217L, I253V, V266K, S332N, K333Q		
HBV33_2	795 050	18	D7A, N122H, M129L, V163I, R217L, I253V, V266I, R343W		
HBV42_2	707 142	64	D7V, I53L, S109P, L115V, N122H, Q125E, M129L, V142D, V163I, R217L, I253V		
HBV62_2	93 167	90	D7A, Y126H, M129L, V163I, **L180M, M204V**, R217L, L229M, I253V, P325A, R343E	**L180M + M204V**	LAM:R<br>ADV: S<br>ETV: I<br>TDF: S
HBV85_2	43	9	P108S, L128M, V278I, I290V, G292D		
HBV88_2		44	S109P, N122H, Q125E, M129L, V163L, R217L		
HBV95_2	41 867	32	D7V, I53L, S109P, N122H, Q125E, M129L, V163I, R217L, I253V		
HBV103_2	85 680	8	D7V, I53L, S109P, N122H, Q125E, M129L, V163I, **V173L, L180M, M204V**, R217L, I253V, R343W	**V173L + L180M + M204V**	LAM:R<br>ADV: I<br>ETV: R<br>TDF: S

Supplementary Table S3: Accession numbers of pol sequences on deposited on GenBank

Sequence identifier	Accession number
1. BankIt2541720 KP53-9X_pol	OM389851
2. BankIt2541720 KP53-10_pol	OM389852
3. BankIt2541720 KP53-103-2POL_pol	OM389853
4. BankIt2541720 KP53-103_pol	OM389854
5. BankIt2541720 KP53-110_pol	OM389855
6. BankIt2541720 KP53-111-2-X_pol	OM389856
7. BankIt2541720 KP53-111_pol	OM389857
8. BankIt2541720 KP53-112_pol	OM389858
9. BankIt2541720 KP53-128_pol	OM389859
10. BankIt2541720 KP53-130_pol	OM389860
11. BankIt2541720 KP53-134_pol	OM389861
12. BankIt2541720 KP53-136_pol	OM389862
13. BankIt2541720 KP53-139_pol	OM389863
14. BankIt2541720 KP53-144_pol	OM389864
15. BankIt2541720 KP53-145_pol	OM389865
16. BankIt2541720 KP53-146_pol	OM389866
17. BankIt2541720 KP53-14X_pol	OM389867
18. BankIt2541720 KP53-150_pol	OM389868
19. BankIt2541720 KP53-18-2_pol	OM389869
20. BankIt2541720 KP53-18POL_pol	OM389870
21. BankIt2541720 KP53-23_pol	OM389871
22. BankIt2541720 KP53-33-2_pol	OM389872
23. BankIt2541720 KP53-33_pol	OM389873
24. BankIt2541720 KP53-34_pol	OM389874
25. BankIt2541720 KP53-40_pol	OM389875
26. BankIt2541720 KP53-42-2_pol	OM389876

27. BankIt2541720 KP53-42POL_pol	OM389877
28. BankIt2541720 KP53-47_pol	OM389878
29. BankIt2541720 KP53-54_pol	OM389879
30. BankIt2541720 KP53-57_pol	OM389880
31. BankIt2541720 KP53-62-2POL_pol	OM389881
32. BankIt2541720 KP53-67_pol	OM389882
33. BankIt2541720 KP53-69POL_pol	OM389883
34. BankIt2541720 KP53-70POL_pol	OM389884
35. BankIt2541720 KP53-72_pol	OM389885
36. BankIt2541720 KP53-82_pol	OM389886
37. BankIt2541720 KP53-85-2POL_pol	OM389887
38. BankIt2541720 KP53-85_pol	OM389888
39. BankIt2541720 KP53-87_pol	OM389889
40. BankIt2541720 KP53-88-2-X_pol	OM389890
41. BankIt2541720 KP53-88POL_pol	OM389891
42. BankIt2541720 KP53-92_pol	OM389892
43. BankIt2541720 KP53-95-2_pol	OM389893
44. BankIt2541720 KP53-95_pol	OM389894
